# Gene Fusions as Potential Therapeutic Targets in Soft Tissue Sarcomas

**DOI:** 10.3390/biom15060904

**Published:** 2025-06-19

**Authors:** Qiongdan Zheng, Tong Wang, Zijian Zou, Wenjie Ma, Zirui Dong, Jingqin Zhong, Wanlin Liu, Yu Xu, Tu Hu, Wei Sun, Yong Chen

**Affiliations:** 1 Department of Musculoskeletal Oncology, Fudan University Shanghai Cancer Center, Shanghai 200032, China; 24111230085@m.fudan.edu.cn (Q.Z.); 23211230028@m.fudan.edu.cn (T.W.); 22211230037@m.fudan.edu.cn (Z.Z.); 18301050153@fudan.edu.cn (W.M.); 18301050200@fudan.edu.cn (Z.D.); 21211230035@m.fudan.edu.cn (J.Z.); liuwanlin@gt.cn (W.L.); xuyudaniel@gmail.com (Y.X.); zzuhutu@sjtu.edu.cn (T.H.); 2 Department of Oncology, Shanghai Medical College, Fudan University, Shanghai 200032, China

**Keywords:** soft tissue sarcoma, gene fusion, targeted therapeutics

## Abstract

Though having been discovered in one third of sarcomas, gene fusions are less studied in their roles as potential therapeutic targets, making conventional modalities the mainstream treatment options for sarcoma patients. Recent decades have witnessed encouraging progress in basic research delving into mechanisms underlying how gene fusions drive sarcomas; nevertheless, further translation to clinical application fails to keep abreast with the advances achieved in basic science. In this review, we will focus on key chromosomal translocation-driven sarcomas defined by characteristic hallmark fusion oncoproteins, including Ewing sarcoma with EWSR1–FLI1/ERG fusion, epithelioid hemangioendothelioma with WWTR1–CAMTA1/YAP1–TFE1 fusion, and others, to discuss the potential of directly targeting these fusion proteins as therapeutic targets in preclinical and clinical contexts.

## 1. Introduction

Soft tissue sarcoma is a heterogeneous group of malignant tumors that originate from mesenchymal cells such as fat, fibrous tissues, histiocytes, vascular cells, muscles, etc. Approximately one third of sarcomas harbor chromosomal translocations, resulting in gene fusions that can encode either transcription factors possessing dysregulated transcriptional power, tyrosine kinases with constitutive activation, or autocrine growth factors that consistently provide nutrients for themselves [1]. Some gene arrangements often recur in specific sarcoma types, e.g., SS18–SSX1 in synovial sarcoma and EWSR1–FLI1 in Ewing sarcoma, making these hallmark gene fusions ideal biomarkers in diagnosing and monitoring minimal residual diseases [2]. Therefore, gene fusions have been an integral part of the clinical management of translocation-harbored sarcomas.

Compared with employing hallmark gene fusions as diagnostic tools and recurrence surveillance in clinical practice, targeting these fusion oncoproteins as therapeutic approaches always lags behind, even though gene fusions have been identified in an explosive mode by virtue of advances in detecting techniques [2]. Though the past few decades have witnessed the treatment consensus of applying tyrosine kinase inhibitors, e.g., sorafenib, pazopanib, and regorafenib, in some sarcomas endorsed by NCCN guidelines, they are general inhibitors instead of specifically targeted ones. Furthermore, despite the fact that there are breakthroughs in utilizing FDA-approved larotrectinib [3] and entrectinib [4] in NTRK gene fusion-positive sarcomas and selpercatinib in RET gene fusion-positive sarcomas [5], a significant step towards individualized precision medicine, compared with a large pool of sarcomas harboring fusions, more progress is being expected. As a consequence, in current clinical practice, mainstay treatment modalities are still conventional, with surgical resection being the first option if there is no distant metastasis, together with chemotherapy and radiotherapy as neoadjuvant, adjuvant, or systemic therapy.

The difficulties underlying designing targeted therapeutics lie in the fragmentary knowledge of the mechanisms by which these fusion oncoproteins drive sarcomagenesis, though we have a much clearer view of their structures and functions. In retrospect, a majority of FDA-approved targeted cancer drugs exploit overactivated receptor tyrosine kinases triggering aberrant signaling cascades (aforementioned NTRK- and RET-involved fusions) or oversecreted growth factors stimulating environmental components (colony-stimulating factor 1, CSF1R, which will be discussed later in this review) generated by gene fusions as druggable vulnerabilities. However, for a large portion of recombinant genes, they usually follow the fusion modes of transcription factor fused with transcription factor or transcription activator, hence substantially empowering transcriptional effects, disrupting biological networks, and enabling oncogenic potential. Despite the complexity, researchers have strived to employ these fusion components as druggable targets, nevertheless impeded by mortal side effects since these transcription factors are highly involved in cell physiology.

In this review, we will focus on some important sarcomas carrying hallmark gene fusions by category and discuss potential employment of these fusions as potential targeted therapeutics, which may shed light on a more precise treatment in these rare tumors.

## 2. EWSR1–FLI1/ERG as Targets in Ewing Sarcoma

Ewing sarcoma is a bone and soft tissue tumor characterized by morphologically small round blue cells and a molecular fusion gene between an RNA-binding protein FET family member (EWSR1, FUS, TAF15, etc.) and an ETS family transcription factor, most commonly EWSR1–FLI1 resulting from chromosomal translocation t(11;22)(q24;q12), present in around 85% of Ewing sarcomas [6]. Other chimeric fusions include EWSR1–ERG [7], and, rarely, with ETV family members. Taking EWSR1–FLI1 as an example, EWSR1 is ubiquitously expressed in cells with multiple biological functions while FLI1, as a proto-oncogene, transcribes genes regulated in normal cells but loses its transcription control once the 3′ C-terminal DNA-binding domain is fused with the 5′ N-terminal transcriptional activation domain of EWSR1. Therefore, EWSR1–FLI1 is more like a dysregulated counterpart of the normal transcription factor FLI1 and is considered to be undruggable due to its transcription factor-like quality.

Current standard treatment for relapsed/refractory or metastatic patients is chemotherapy, with alternating cycles of vincristine/doxorubicin/cyclophosphamide and ifosfamide/etoposide (VDC/IE) being the most prescribed treatment plan. In addition, targeted tyrosine kinase inhibitors carbozantinib [8] and regorafenib [9,10] have been tested in multiple relapsed patients. Nevertheless, as Meyers and colleagues state in their report on a phase I/II trial of TK216, an EWSR1–FLI1 protein antagonist, direct targeting of the ES chimera is always the most logical approach [11].

The development of this EWSR1–FLI1 chimera inhibitor originates from Toretsky and colleagues’ finding of an EWS–FLI1 interaction partner, RNA helicase A, back in 2006. Toretsky and colleagues reported that the oncogenic function of EWS–FLI1 can be enhanced by complexing with RNA helicase A, providing rationale for exploiting the interaction between EWS–FLI1 and RNA helicase A as a vulnerability in tumors carrying this hallmark gene fusion [12]. Later in their discovery in 2009, Toretsky’s team proposed YK-4-279, a research compound that blocks EWS–FLI1 binding to RNA helicase A, and they observed that it induced apoptosis and reduced tumor growth [13]. The clinical derivative of YK-4-279 [14], TK216, has been employed in clinical trials owing to these positive effects in preclinical models (NCT02657005, NCT05046314). NCT02657005 is a phase I/II clinical trial designed to evaluate the safety, tolerability, and clinical activity at the recommended phase II dose (RP2D). The trial has been terminated, and the study results were published by Meyers et al. in July 2024. In the report, the efficacy of TK216—though promising in the three responders, including two complete remissions who remained disease-free four years after study entry—was nonetheless considered limited and insufficient to warrant further evaluation under the treatment protocol used in the trial [11].

In addition to RNA Helicase A, which interacts with the EWS–FLI1 fusion protein and is being evaluated in clinical trials as a druggable vulnerability, histone lysine demethylase (LSD1) is another interaction partner of FET-fusion sarcoma (including Ewing sarcoma) with therapeutic potential for targeted disruption [14]. Sankar et al. demonstrated that a non-competitive LSD1 inhibitor, HCI2509, disrupted transcriptional profiles and malignant characteristics of EWS/FLI- and EWS/ERG-containing Ewing sarcoma [15] in preclinical evaluations. INCB059872 (NCT03514407) and seclimastat (NCT03600649) are both LSD1 inhibitors as interventions in clinical trials for Ewing sarcoma.

**Figure 1:**
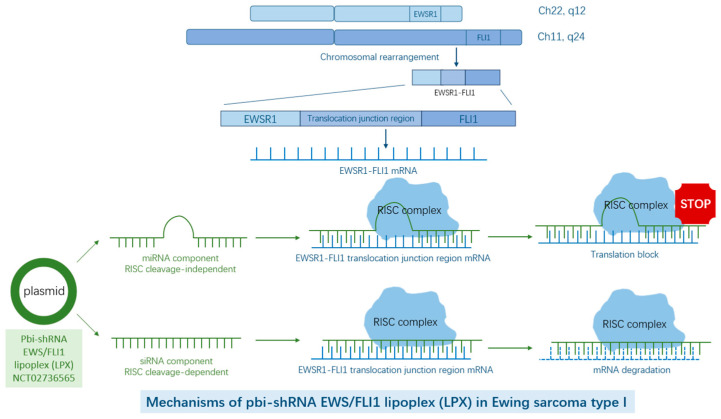
It would be better to change the figure caption to “Pbi-shRNA EWS/FLI lipoplex (LPX), a bi-functional plasmid construct designed to degrade the mRNA transcribed from the EWSR1–FLI1 translocation junction region, is currently in phase I clinical trial”.

Furthermore, Pbi-shRNA EWS/FLI1 lipoplex (LPX) is a functional plasmid construct (Figure 1) that breaks the translocation junction region of EWS–FLI1 and target mRNAs [16], and a phase I clinical trial (NCT02736565) has been completed. More clinical results will need to be released to see if the trial can be moved to further phases.

## 3. WWTR1–CAMTA1/YAP1–TFE1 as Targets in Epithelioid Hemangioendothelioma

Epithelioid hemangioendothelioma (EHE) is a rare sarcoma that develops from vascular endothelial cells. It has a propensity for systemic involvement, with the lungs and liver being the most common sites [17].

The name “epithelioid hemangioendothelioma” was first proposed by Weiss and Enziger to refer to an angiocentric tumor with an epithelioid appearance and borderline-to-malignant potential for its possession of both hemangioma and angiosarcoma features [18], while before that, the tumor was mistaken for a carcinoma. EHE cells are less disorganized and dedifferentiated than those in angiosarcoma, which can be quite disorganized and pleomorphic.

**Figure 2:**
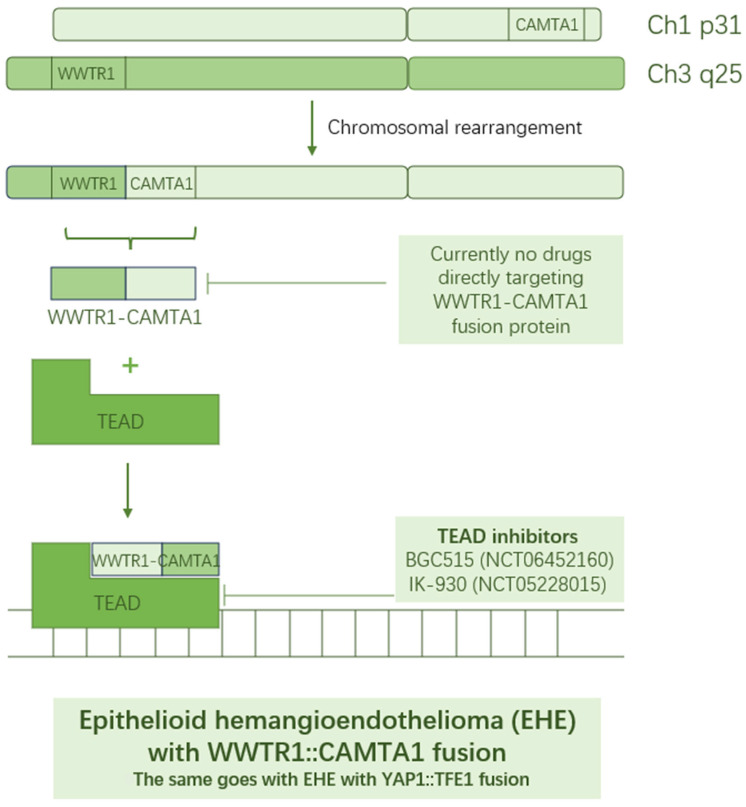
Mechanism of WWTR1–CAMTA1 fusion protein driving epithelioid hemangioendothelioma (EHE) development and TEAD, a protein interacting with WWTR1–CAMTA1, may be a potential target for controlling EHE.

As the name indicates, EHE with the WWTR1–CAMTA1 fusion is named after its characteristic gene fusion, WWTR1–CAMTA1, due to a gene arrangement between chromosome 1 and chromosome 3, denoted by t(1;3)(p36;q25), constituting 90% of EHE. WWTR1 encodes a transcription factor highly involved in the Hippo pathway, which is critical for endothelial cell growth, whereas CAMTA1 contains a transcriptional activation domain. Therefore, the aberrantly fused gene has potent nuclear localization and transcriptional functions, which cause a dysregulated angiogenic program in vascular endothelial cells. Similar to other fused transcription factors, WWTR1–CAMTA1 is difficult to target directly due to its lack of enzymatic or kinase activity. Consequently, targeting essential interaction partners of the fusion protein remains the primary therapeutic approach. Driskill and colleagues identified TEAD as a critical transcriptional partner of WWTR1–CAMTA1 to initiate oncogenic events in EHE [19]. They found that disrupting the interaction between WWTR1–CAMTA1 and TEAD, or ectopically expressing a dominant-negative TEAD, can inhibit WWTR1–CAMTA1-induced EHE. This discovery has provided a rationale for advancing TEAD inhibitors to clinical evaluation in EHE. BGC515 (NCT06452160) and IK-930 (NCT05228015) are both TEAD inhibitors (Figure 2) under investigation in solid tumors with a dysregulated Hippo pathway, including EHE. Moreover, there has been a report on CAMTA1 fusion-related activation of the MEK signaling pathway [20], justifying the employment of the MEK inhibitor, trametinib, in EHE treatment (NCT03148275)

Similarly, EHE with YAP1–TFE1 fusion (found in 10% of EHE) is defined by its hallmark gene fusion YAP1-TFE1, with a similar mode of transcription regulator (YAP1) paired with a transcription factor (TFE1) to WWTR1–CAMTA1. The transcriptional potential of TFE1 is greatly boosted by its promoter region being juxtaposed with YAP1 transactivation domains [21]. This aberrant activation of the Hippo pathway ultimately leads to morphological abnormalities and biological abnormalities. Yuan et al. found that YAP1 interacts with TEAD to amplify Hippo signaling [22]. Clinical trials involving Hippo pathway inhibitors BGC515 (NCT06452160) and IK-930 (NCT05228015) are open to EHE patients with no specific requirement for the type of gene fusion that they harbor.

## 4. FUS/EWSR1–DDIT3 as Targets in Myxoid Liposarcoma

Myxoid liposarcoma (MLPS) is characterized by pathognomonic chromosomal translocations t(12;16)(q13;p11), resulting in the FUS–DDIT3 fusion oncoprotein, which has been detected in more than 90% of MLPS [23], while in a smaller portion (reportedly around 3%) of cases, EWSR1–DDIT3 is fused as a result of the gene arrangement t(12;22)(q13;q12) [24]. FUS-DDIT3 contains N-terminal regions of FUS and almost the full length of DDIT3, with the latter being the critical dysregulated transcription driver in MLPS [24]. Zullow and colleagues demonstrated that the FUS–DDIT3 fusion protein drives the development of myxoid liposarcoma by sequestering the adipogenic transcription factor, CEBPB, and thus disrupting BAF-mediated gene targeting, leading to oncogenic gene transcription [25]. Trautmann et al. found that FUS–DDIT3 fusion oncoprotein induces aberrant activation of IGF–1R/PI3K/Akt signaling in MLPS [26]. Furthermore, Trautmann and colleagues revealed that FUS–DDIT3 promotes YAP1 activity and hence Hippo signaling [27]. These mechanisms provide a rationale to target the fusion oncoproteins FUS/EWSR1–DDIT3 directly or indirectly through downstream signaling, such as IGF1R or Hippo signal transduction, as therapeutics.

Consistent with previously described sarcoma subtypes, FUS/EWSR1–DDIT3 fusions are challenging to target directly with existing technologies due to their transcription factor nature. Therefore, transcriptional interaction partners or downstream signaling pathways such as IGF1R and Hippo signaling hold more promise as targeted therapeutic approaches. Currently, most therapeutic interventions in clinical trials either disrupt the interaction between FUS/EWSR1-DDIT3 fusion proteins and their target mRNAs, inhibit downstream signaling caused by dysregulated transcription, or target specific antigens expressed on the tumor cell surface. Seclidemstat, a lysine-specific demethylase 1 (LSD1) inhibitor, reportedly blocking the transcription function of fusion oncoproteins involving FET family members (FUS and EWSR1 are both FET family transcription factors) [14], is now under active clinical trial (NCT03600649). A phase I clinical trial on cixutumumab, an IGF1R inhibitor that suppresses upregulated IGF signaling in MLPS, was tested together with a conventional chemotherapy drug, doxorubicin (NCT00720174). Mirdametinib, a MEK inhibitor, is under a phase Ib/II study in combination with palbociclib in liposarcoma (NCT06843967). Sirolimus, an mTOR inhibitor, was studied with cyclophosphamide in metastatic or unresectable MLPS (NCT02821507). A phase I/II clinical trial on combination therapy of vismodegib, an FDA-approved hedgehog signaling inhibitor, with a gamma-secretase inhibitor, RO4929097, a NOTCH signaling-targeted drug, has been completed (NCT01154452). Moreover, targeted therapies against specific antigens expressed on tumor cell surfaces are also being evaluated, such as gene-modified T-cell therapies targeting MAGE-A4 (NCT04044768, NCT03132922) and NY-ESO-1 (NCT02992743, NCT03399448, etc.).

## 5. SS18-SSX1/2/4 as Targets in Synovial Sarcoma

Synovial sarcoma constitutes 5–10% of all soft tissue sarcomas and is characterized by its unique pathognomonic chromosomal translocations between SS18 and SSX1/2, and less commonly SSX4. Synovial sarcoma not only originates from the synovium but can also derive from primitive mesenchymal cells. Therefore, it is not confined to the joints and can occur anywhere in the body. There are three histological subtypes: monophasic/spindle cell, biphasic, and poorly differentiated [28].

**Figure 3:**
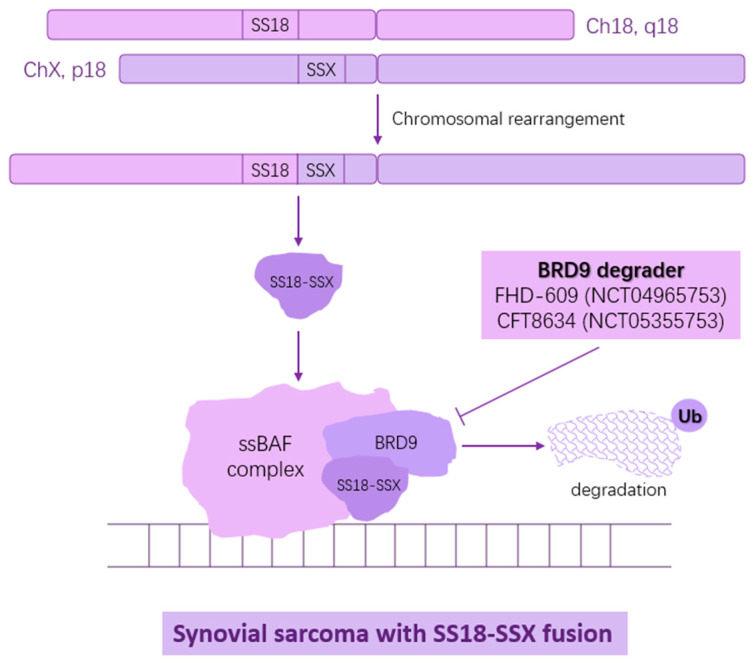
Mechanism of the fusion protein SS18–SSX driving synovial sarcoma development and BRD9, a protein interacting with SS18–SSX, may be a potential target for controlling synovial sarcoma.

The incorporation of the SS18–SSX fusion oncoprotein into the SWI/SNF complex activates a synovial sarcoma transcriptional signature, thereby driving sarcomagenesis [29,30]. As a result, the interaction between SS18–SSX and SWI/SNF presents a promising target for therapeutic intervention. Brien et al. reported that targeted degradation of BRD9, a component of the SWI/SNF complex found in synovial sarcoma, can inhibit tumor progression [31], which lays the foundation for clinical trials of the BRD9 degraders FHD-609 (NCT04965753) and CFT8634 (NCT05355753) (Figure 3) in SS; however, they were terminated owing to a lack of sufficient efficacy as single agents.

In addition to inhibiting transcriptional interaction components, dysregulated downstream signaling pathways also represent ideal therapeutic targets. Wnt signaling has been shown to be overactivated by oncogenic fusion proteins in synovial sarcoma [32]. There has been research revealing that frizzled homolog 10 (FZD10), a Wnt signaling receptor, is overexpressed in a majority of synovial sarcomas while absent in most normal tissues, which justifies the clinical trial of a radiolabeled monoclonal antibody against FZD10 (NCT01469975), though less toxic radioisotopes might be a better option as stated by the results report [33]. Furthermore, the aberrant expression of receptor tyrosine kinases such as VEGFR and PDGFR, driven by fusion oncoproteins in synovial sarcoma, has prompted clinical trials of multi-kinase inhibitors (NCT03016819).

## 6. COL6A3-CSF1 as Target in Malignant Tenosynovial Giant Cell Tumor

Malignant tenosynovial giant cell tumor (mTSGCT) is an extremely rare sarcoma that arises from the synovium of joints and tendon sheaths. mTSGCT can be either the sarcomatous area within benign TSGCT or a de novo growth arising from multiple recurrences of benign TSGCT. It follows a natural history of great potential for local infiltration and distant metastasis [34]. West and colleagues were the first researchers to define mTSGCT and characterize its hallmark fusion gene, whereas previously, TSGCT had been considered a benign lesion (pigmented villonodular synovitis was used then to refer to mTSGCT and is now obsolete). They revealed that TSGCT is actually a polyclonal and heterogeneous tumor consisting of a small group of neoplastic cells and a major group of non-neoplastic cells recruited by CSF1 (colony-stimulating factor-1) released from this minority population as a result of their hallmark chromosomal translocation COL6A3–CSF1 [35].

**Figure 4:**
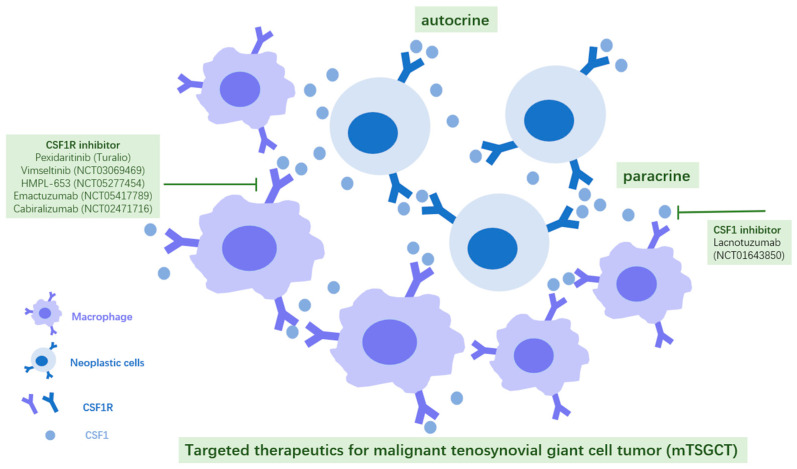
Mechanism of tenosynovial giant cell tumor formation and targeted therapeutics in clinical trials.

There have not been any drugs that can directly break down the COL6A3–CSF1 transcript or oncoprotein. However, considering the fact that the major TSGCT components are mono/macrophage lineages expressing CSF1R, outweighing the small portion of cancer cells with CSF1 autocrine and paracrine, CSF1R inhibitors have been designed to selectively target TSGCT non-neoplastic components to shrink tumors (Figure 4). CSF1R inhibitors are more effective in benign TSGCTs than in malignant ones, as benign tumors are more dependent on CSF1-mediated signaling. Pexidaritinib (Turalio) has been approved by the FDA in August 2019 for symptom control in TSGCT. Though malignant tumors are less dependent on CSF1 stimulation, exhibit more autonomous growth, and their treatment is sometimes further complicated by additional genetic alterations, given that both benign and malignant TSGCTs share the same underlying fusion, CSF1R inhibition remains a central concept in therapeutic development. There are currently two medications in clinical trials specifically for advanced TSGCT patients: vimseltinib (NCT03069469) and HMPL-653 (NCT05277454). Several other promising drugs with similar mechanisms in clinical trials include emactuzumab (NCT05417789), cabiralizumab (NCT02471716), etc. Moreover, it is worth mentioning that lacnotuzumab, a monoclonal antibody that directly targets CSF1, is in clinical trial (NCT01643850) (Figure 4). These drugs may be generalized to malignant cases due to the same mechanisms in treating this broader type of disease.

## 7. PAX3/7–FOXO1 as Targets in Alveolar Rhabdomyosarcoma

Alveolar rhabdomyosarcoma (ARMS) is the second most frequently occurring RMS, comprising around 25% of all RMS [36]. It histologically shows cell nests composed of primitive cells with skeletal muscle differentiation separated by fibrovascular septa, giving an “alveolar” pattern. Molecular analyses have discovered that the oncoproteins PAX3–FOXO1 and PAX7–FOXO1, produced by chromosomal translocations t(2;13)(q36;q14) and t(1;13)(p36;q14), respectively, are the most common mechanisms that drive the oncogenesis of ARMS [37]. Both fusion components PAX3/7 and FOXO1 are transcription factors, with PAX family members possessing myogenic effects. When juxtaposed, DNA-binding domains in PAX3/7 are paired with transcription-activating domain in FOXO1, conferring these fused genes boosted transcriptional effects that result in a dysregulated myogenic expression program [38]. The strong causal relationship between PAX3/7–FOXO1 fusion oncoproteins and alveolar rhabdomyosarcoma provides the rationale to employ therapeutic approaches targeting these fusion proteins. However, there are currently no direct PAX3/7–FOXO1-targeted drugs in clinical trials due to inconclusive information on the ideal molecular structures of blocking sites or the complex protein interactions that these fusion proteins are involved in [39]. Singh and colleagues reported that PAX3–FOXO1 interacts with KDM4B in driving the oncogenesis of RMS [40]. Therefore, it is encouraging that KDM4B may be a therapeutic vulnerability in PAX–FOXO1-positive RMS, although it has not yet advanced to clinical trials. Moreover, Martins et al. summarized that IGF1R signaling is activated in PAX3/7–FOXO1 rhabdomyosarcoma [41], probably through the dysregulated transcription of the oncogenic chimeric proteins. Currently, there are several IGF1R inhibitors in clinical trials, including ganitumab (NCT03041701) and cixutumumab (NCT01055314, NCT01614795) as components in combination therapies. Wan and colleagues reported rebound activation of YES, an SRC-family tyrosine kinase, when an IGF-1R inhibitor was applied in alveolar and embryonal RMS cell lines; therefore, this research provides the background for the clinical trial combining IGF-1R inhibitor ganitumab with the Src-family kinase inhibitor dasartinib to prevent resistance to using IGF-1R alone (NCT03041701). Results reported in June 2022 showed that the combination therapy may delay resistance to IGF-1R inhibitors in RMS [42]. With a similar treatment concept, a clinical trial of the combination therapy of the IGF-1R inhibitor cixutumumab and the mTOR inhibitor temsirolimus was designed to target potential rebound activation of mTOR signaling when the IGF-1R inhibitor is applied alone (NCT01614795).

## 8. Other Rare Soft Tissue Sarcomas with Hallmark Fusion Proteins and Their Potential Targeted Therapeutics

Malignant solitary fibrous tumor (SFT) is characterized by recurrent inversion of chromosome 12q13, leading to gene fusion NAB2-STAT6. Normally, NAB2 is a transcriptional repressor, while upon fusion, its repressor domain is truncated by STAT6, a transcriptional transactivator, resulting in a chimeric transcription factor located in the nucleus that drives the oncogenesis of SFT [43]. It is reported that NAB2–STAT6 has control over the expression of proangiogenic factors VEGFA, bFGF, etc., which is consistent with the highly vascularized feature that SFT presents. There are several drugs targeting angiogenic tyrosine receptor kinases under study or having completed clinical trials with results. Axitinib is a drug specifically targeting VEGFR2 and PDGFRB, thereby inhibiting the vascularization of SFT; it has been evaluated for advanced SFT patients in the trial NCT02261207. In addition, Pazopanib is another anti-angiogenic drug that specifically targets VEGFR and has been evaluated in a phase II clinical trial for SFT patients (NCT02066285). Although there are no drugs directly targeting this pathognomonic fusion oncoprotein found in Clinicaltrial.gov at present, antisense oligonucleotides (ASOs) that bind to the untranslated region (UTR) of the C-terminal STAT6 transcript, resulting in suppressed expression of NAB–STAT6 transcripts, provides encouraging evidence for future targeted therapy design [44].

Alveolar soft part sarcoma is characterized by pathognomonic chromosomal translocation der(17)t(X;17)(p11.2;q25), leading to the ASPSCR1–TFE3 fusion oncoprotein [45]. Despite the fact that its hallmark gene fusion is well characterized, targeted drugs reconstructing disordered signaling driven by the aberrant fusion have not yet been devised. Tanaka and colleagues discovered in their ASPSCR1–TFE3-driven mouse model that a transcriptional target of the hallmark gene fusion ASPSCR1–TFE3, GPNMB (which encodes glycoprotein nmb), was highly expressed to facilitate tumor invasion and migration; while silencing GPNMB could inhibit these malignant characteristics [46], supporting the idea that GPNMB may be a druggable target. Moreover, due to a lack of panoramic view of ASPSCR1-TFE3 function besides fragmentary knowledge of upregulated receptor tyrosine kinase signaling pathways, most drugs in clinical trials or proposals are general inhibitors that focus on suppressing cascade amplification provoked by stimulation of these receptors or intracellular kinases. Tsuda and colleagues reported that gene fusions involving TFE3 can activate MET signaling; hence, they proposed that MET inhibitors may be potential candidates for managing alveolar soft part sarcoma [47]. Another example would be a pan-VEGFR inhibitor, cediranib, in clinical trials (NCT01391962, NCT00942877, NCT01337401). Because alveolar soft part sarcomas are highly vascular tumors, the anti-angiogenic agent cediranib has been employed to study its effects in this disease.

Spindle cell rhabdomyosarcoma (SpRMS) is a rare variant of rhabdomyosarcoma characterized by its fascicular spindle cell morphology. Further subclassification is based on genetic alterations. Congenital or infantile SpRMS harbors chromosomal rearrangements VGLL2–CITED2 or NCOA2–CITED2, which may indicate a favorable prognosis [48]. Both VGLL2/NCOA2 and CITED2 are transcription coactivators, while the mechanisms by which these fusion oncoproteins leverage sarcomagenesis are poorly understood. Yamaguchi concluded that VGLL2-involved fusion genes interact with TEAD to regulate gene expression [49], similar to the aforementioned mechanisms employed by EHE bearing specific fusions. Consequently, we can probably assume that TEAD inhibitors may as well be applied in SpRMS patients to interfere with the interaction between pathognomonic fusion proteins and their target mRNAs. The MYOD1 mutant subset of SpRMS is associated with sclerosing morphology and has unfavorable outcomes compared with the other two subtypes of SpRMS [50]. Intraosseous spindle cell rhabdomyosarcoma is characterized by fusion genes consisting of TFCP2 or NCOA2 as one of the components, whereas their paired partners can be ESWR1, FUS (with TFCP2), and MEIS (with NOCA2). Characterization of these fusion proteins will shed light on targeted therapy design for these druggable sites, although currently, most clinical trials recruiting SpRMS patients focus on broadly targeted therapies or chemotherapy.

Phosphaturic mesenchymal tumor (PMT) is a very rare type of mesenchymal tumor that is associated with osteomalacia as a result of overexpression of fibroblast growth factor 23 (FGF23). Most PMTs harbor the FN1–FGFR1 gene fusion that expresses the receptor tyrosine kinase FGFR. When overstimulated by FGF23, renal phosphate reabsorption is inhibited [51]. Hartley and colleagues reported a case of tumor-induced osteomalacia caused by PMT, in which the patient participated a clinical trial that studied infigratinib, an FGFR1-3 inhibitor (NCT02160041), and experienced a dramatic response, with FGF23 levels decreasing to almost one-tenth in the first 24 h and returning to normal after 2 weeks [52]. Although the patient ultimately died due to intermittent discontinuation caused by side effects, the case presents evidence for applying therapeutics targeting the pathognomonic gene fusion. Furthermore, there are clinical trials studying the monoclonal antibody burosumab, which directly targets FGF23 (NCT05357573, NCT02722798).

Clear cell sarcoma is characterized by specific chromosomal rearrangements, EWSR1–ATF1 or EWSR1–CREB1. It exhibits melanocytic features due to occupation of the MITF promoter with EWS–ATF1, resulting in MITF expression, similar to canonical MITF expression in melanocyte-stimulating hormone signaling [53]. Moreover, Davis and colleagues revealed that MITF activated by EWS–ATF1 can further activate the transcription of c-MET, a receptor tyrosine kinase, while c-MET blockade or ligand (hepatocyte growth factor) neutralization can inhibit cell growth [54]. By this means, they provided a basis for potential benefits from c-MET inhibitors. In terms of direct-targeted drugs to the fusion oncoprotein, Mae and colleagues reported that HDAC inhibitor vorinostat can suppress transcription driven by EWSR1–ATF1 through inhibiting BRD4, a protein at the fusion gene promoter region [55], suggesting a novel fusion oncoprotein-targeted mode that might be employed in the future.

Desmoplastic small round cell tumor (DSRCT) is characterized by aberrant chimeric oncoproteins EWSR1–WT1. Magrath and colleagues revealed that, unlike the transcription patterns of other fusion proteins, ESWR1–WT1 can mediate transcription through direct binding to target mRNAs. It can bind to the CCND1 (which encodes cyclin D1, a subunit of CDK4/6) promoter and stimulate tumor growth [56]. Their findings provide rationale for employing CDK4/6 inhibitors as potential therapeutics for DSRCT. Furthermore, the clinical trial studying seclidemstat, an LSD1 inhibitor that disrupts transcription of fusions containing the FET family, has also aimed to enroll DSRCT patients (NCT03600649). Currently, there are no direct-targeted drugs for oncogenic ESWR1–WT1.

Extraskeletal myxoid chondrosarcoma (EMC) is characterized by hallmark chromosomal rearrangements involving NR4A3, leading to constitutive expression of NR4A3. The most common fusion partners include EWSR1 or TAF15, with others reviewed elsewhere [57]. Given that these fusion partners are all FET family members, seclidemstat, as previously mentioned in myxoid liposarcoma harboring EWSR1/FUS–DDIT3 chromosomal translocations, may have promising effects in extraskeletal myxoid chondrosarcoma, considering that seclidemstat, an LSD1 inhibitor, can block transcription of fusions involving FET family members (NCT03600649). Currently, there have not been any drugs targeting NR4A3 in clinical trials. Pazopanib and apatinib have been reported to inhibit receptor tyrosine kinases, whose activities are greatly increased by dysregulated pathway programming caused by NR4A3-involved fusion oncoproteins. Stacchiotti and colleagues reported the results of a pazopanib clinical trial in patients with advanced extraskeletal myxoid chondrosarcoma (NCT02066285). They observed clinically meaningful effects, which may provide evidence for considering pazopanib as a treatment option.

A list of clinical trials on targeted therapies in sarcomas with hallmark gene fusions is presented in the following table (Table 1).

**Table 1:** Sarcomas with hallmark gene fusions and targeted therapies in clinical trials.

Clinical Trials	NCT Number	Trial Phase	Status	Interventions
**Myxoid Liposarcoma**				
Clinical Trial of SP-2577 (Seclidemstat) in Patients with Relapsed or Refractory Ewing or Ewing-related Sarcomas	NCT03600649	Phase I	Active, not recruiting	Seclidemstat, a LSD1 inhibitor
Cixutumumab and Doxorubicin Hydrochloride in Treating Patients with Unresectable, Locally Advanced, or Metastatic Soft Tissue Sarcoma	NCT00720174	Phase I	Completed	Cixutumumab, IGF-1R inhibitor, in combination with chemotherapy drugs
Vismodegib and Gamma-Secretase/Notch Signalling Pathway Inhibitor RO4929097 in Treating Patients with Advanced or Metastatic Sarcoma	NCT01154452	Phase I/II	Completed	Vismodegib, Hedgehog signaling inhibitor, together with RO4929097, Notch signaling inhibitor
Spearhead 1 Study in Subjects with Advanced Synovial Sarcoma or Myxoid/Round-Cell Liposarcoma	NCT04044768	Phase II	Recruiting	Car-T cell targeting MAGE-A4
MAGE-A4^c1^^o^^32^T for Multi-Tumor	NCT03132922	Phase I	Active, not recruiting	Car-T cell targeting MAGE-A4
Letetresgene Autoleucel Engineered T Cells in NY-ESO-1 Positive Participants with Advanced Myxoid/Round-Cell Liposarcoma	NCT02992743	Pilot study	Completed	Car-T cell targeting NY-ESO-1
NY-ESO-1-redirected CRISPR (TCRendo and PD1) Edited T Cells (NYCE T Cells)	NCT03399448	Phase I	Completed	Car-T cell targeting NY-ESO-1
**Malignant tenosynovial giant cell tumor (mTSGCT)**				
Study of Vimseltinib (DCC-3014) in Patients with Advanced Tumors and Tenosynovial Giant Cell Tumor	NCT03069469	Phase I/II	Active, not recruiting	Vimseltinib, CSF1R inhibitor
Clinical Study of HMPL-653 in Treatment of Advanced Malignant Solid Tumors and TGCT	NCT05277454	Phase I	Recruiting	HMPL-653, CSF1R inhibitor
Study of Emactuzumab for Tenosynovial Giant Cell Tumor (TGCT) (TANGENT)	NCT05417789	Phase III	Recruiting	Emactuzumab, CSF1R inhibitor
Study of Cabiralizumab in Patients with Pigmented Villonodular Synovitis/Diffuse Type Tenosynovial Giant Cell Tumor (FPA008-002)	NCT02471716	Phase I/II	Completed	Cabiralizumab, CSF1R inhibitor
MCS110 in Patients with Pigmented Villonodular Synovitis (PVNS)	NCT01643850	Phase II	Completed	MCS110, CSF1 inhibitor
**Epithelioid hemangioendothelioma**				
A Study of BGC515 Capsules in Subjects with Advanced Solid Tumors	NCT06452160	Phase I	Recruiting	BGC515, TEAD inhibitor
Oral TEAD Inhibitor Targeting the Hippo Pathway in Subjects with Advanced Solid Tumors	NCT05228015	Phase I	Active, not recruiting	IK-930, TEAD inhibitor
Trametinib in Treating Patients with Epithelioid Hemangioendothelioma That is Metastatic, Locally Advanced, or Cannot Be Removed by Surgery	NCT03148275	Phase II	Completed	Trametinib, MEK inhibitor
**Alveolar rhabdomyosarcoma**				
Insulin-like Growth Factor 1 Receptor (IGF-1R) Antibody AMG479 (Ganitumab) in Combination with the Src-Family Kinase (SFK) Inhibitor Dasatinib in People with Embryonal and Alveolar Rhabdomyosarcoma	NCT03041701	Phase I/II	Terminated	Ganitumab, IGF-1R inhibitor, in combination with Dasatinib, Src-family kinase inhibitor
Temozolomide, Cixutumumab, and Combination Chemotherapy in Treating Patients with Metastatic Rhabdomyosarcoma	NCT01055314	Pilot study	Completed	Cixutumumab, IGF-1R inhibitor, in combination with chemotherapy drugs
Cixutumumab and Doxorubicin Hydrochloride in Treating Patients with Unresectable, Locally Advanced, or Metastatic Soft Tissue Sarcoma	NCT00720174	Phase I	Completed	Cixutumumab, IGF-1R inhibitor, in combination with chemotherapy drugs
Cixutumumab and Temsirolimus in Treating Younger Patients with Recurrent or Refractory Sarcoma	NCT01614795	Phase II	Completed	Cixutumumab, IGF-1R inhibitor, in combination with Temsirolimus, mTOR inhibitor
**Phosphaturic mesenchymal tumor**				
BGJ398 for Patients with Tumors with FGFR Genetic Alterations (CBGJ398XUS04)	NCT02160041	Phase II	Terminated	BGJ398, FGFR inhibitor
BGJ398 for the Treatment of Tumor-Induced Osteomalacia	NCT03510455	Phase II	Terminated	BGJ398, FGFR inhibitor
Study to Assess the Safety, Pharmacokinetics and Efficacy of KRN23 in Adult Chinese Patients With TIO	NCT05357573	Phase IV	Completed	KRN23, FGF23 monoclonal antibody
A Study of KRN23 in Subjects with Tumor-Induced Osteomalacia or Epidermal Nevus Syndrome	NCT02722798	Phase II	Completed	KRN23, FGF23 monoclonal antibody
**Desmoplastic small round cell tumor**				
Clinical Trial of SP-2577 (Seclidemstat) in Patients with Relapsed or Refractory Ewing or Ewing-related Sarcomas	NCT03600649	Phase I	Active, not recruiting	Seclidemstat, LSD1 inhibitor
**Synovial sarcoma**				
First in Man Study Investigating the Biodistribution, the Safety and Optimal Recommended Dose of a New Radiolabelled Monoclonal Antibody Targeting Frizzled Homolog 10 (SYNFRIZZ)	NCT01469975	Phase I	Terminated	Yttrium 90-radiolabeled OTSA101 (OTSA101-DTPA-90Y), radiolabeled FZD10 inhibitor
Phase I Study of Radiolabeled OTSA101-DTPA in Patients with Relapsed or Refractory Synovial Sarcoma	NCT04176016	Phase I	Terminated	Yttrium 90-radiolabeled OTSA101 (OTSA101-DTPA-90Y), radiolabeled FZD10 inhibitor
FHD-609 in Subjects with Advanced Synovial Sarcoma or Advanced SMARCB1-Loss Tumors	NCT04965753	Phase I	Terminated	FDH-609, sSWI/SNF component BRD9 inhibitor
A Study to Assess the Safety and Tolerability of CFT8634 in Locally Advanced or Metastatic SMARCB1-Perturbed Cancers, Including Synovial Sarcoma and SMARCB1-Null Tumors	NCT05355753	Phase I/II	Terminated	CFT8634, BRD9 degrader
**Alveolar soft part sarcoma**				
Sunitinib or Cediranib for Alveolar Soft Part Sarcoma	NCT01391962	Phase II	Active, not recruiting	Sunitinib or cediranib, multi-RTK inihibitors
Phase II Study of Cediranib (AZD2171) in Patients with Alveolar Soft Part Sarcoma	NCT00942877	Phase II	Active, not recruiting	Cediranib, a pan-VEGFR inhibitor
A Trial of Cediranib in the Treatment of Patients with Alveolar Soft Part Sarcoma (CASPS) (CASPS)	NCT01337401	Phase II	Unknown status	Cediranib, a pan-VEGFR inhibitor
**Extraskeletal myxoid chondrosarcoma**				
Clinical Trial of SP-2577 (Seclidemstat) in Patients with Relapsed or Refractory Ewing or Ewing-related Sarcomas	NCT03600649	Phase I	Active, not recruiting	Seclidemstat, LSD1 inhibitor
Trial of Pazopanib in Patients with Solitary Fibrous Tumor and Extraskeletal Myxoid Chondrosarcoma	NCT02066285	Phase II	Completed	Paopanib, multi-kinase inhibitor
**Ewing sarcoma**				
A Clinical Study of TK216 in Patients with Relapsed or Refractory Ewing’s Sarcoma	NCT05046314	Phase II	Recruiting	TK216, a ETS binding drug
TK216 in Patients with Relapsed or Refractory Ewing Sarcoma	NCT02657005	Phase I/II	Terminated	TK216, a ETS binding drug
A Study of INCB059872 in Relapsed or Refractory Ewing Sarcoma	NCT03514407	Phase I	Terminated	INCB059872, a LSD1 inhibitor
Clinical Trial of SP-2577 (Seclidemstat) in Patients with Relapsed or Refractory Ewing or Ewing-related Sarcomas	NCT03600649	Phase I	Active, not recruiting	Seclidemstat, a LSD1 inhibitor
Pbi-shRNA™ EWS/FLI1 Type 1 LPX in Subjects with Advanced Ewing’s Sarcoma	NCT02736565	Phase I	Completed	Pbi-shRNA EWS/FLI1 lipoplex, a plasmid construct that breaks junction between EWS–FLI1 and target mRNAs

## 9. Conclusions

In this review, we discussed several key sarcoma subtypes characterized by hallmark fusion proteins—such as Ewing sarcoma with EWSR1–FLI1/ERG, epithelioid hemangioendothelioma with WWTR1–CAMTA1/YAP1–TFE1 fusion—and highlighted their clinical implications as targets for therapy. We summarized that current targeted therapeutic strategies primarily focus on disrupting transcriptional interaction partners or inhibiting downstream signaling pathways of fusion oncoproteins, as direct targeting of the fusion proteins remains challenging due to their lack of enzymatic or kinase activity. Furthermore, with the emergence of advanced technologies such as proteolysis-targeting chimeras (PROTACs), molecular adhesive degradation-targeting chimeras (MADTACs), RNA-based therapies, and CRISPR-based therapies, more novel approaches to target fusion oncoproteins are anticipated. Though many of them are still in the conceptual stage or undergoing early-phase clinical evaluation, they represent a promising direction in sarcoma treatment.

## Data Availability

As this manuscript is a review article, no new data were generated or analyzed. All data discussed are sourced from previously published studies, which are appropriately cited in the textt.

## Abbreviations

The following abbreviations are used in this manuscript:

CSF1R	Colony-stimulating factor 1.
MLPS	Myxoid liposarcoma.
LSD1	Lysine specific demethylase 1.
SFT	Solitary fibrous tumor.
ASO	Antisense oligonucleotide.
UTR	Untranslated region.
TSGCT	Tenosynovial giant cell tumor.
EHE	Epithelioid hemangioendothelioma.
ARMS	Alveolar rhabdomyosarcoma.
mTOR	Mammalian target of rapamycin.
SpRMS	Spindle cell rhabdomyosarcoma.
PMT	Phosphaturic mesenchymal tumor.
FGF	Fibroblast growth factor.
EMC	Extraskeletal myxoid chondrosarcoma.
DSRCT	Desmoplastic small round-cell tumor.
OFMT	Ossifying fibromyxoid tumor.
MECA	Myoepithelial carcinoma.
